# Acute effect of hydrogen-rich water on physical, perceptual and cardiac responses during aerobic and anaerobic exercises: a randomized, placebo-controlled, double-blinded cross-over trial

**DOI:** 10.3389/fphys.2023.1240871

**Published:** 2023-12-14

**Authors:** Nidhal Jebabli, Nejmeddine Ouerghi, Wissal Abassi, Fatma Hilal Yagin, Mariem Khlifi, Manar Boujabli, Anissa Bouassida, Abderraouf Ben Abderrahman, Luca Paolo Ardigò

**Affiliations:** ^1^ Research Unit: “Sport Sciences, Health and Movement”, High Institute of Sport and Physical Education of Kef, University of Jendouba, Kef, Tunisia; ^2^ Faculty of Medicine of Tunis, Rabta Hospital, University of Tunis El Manar, LR99ES11, Tunis, Tunisia; ^3^ High Institute of Sport and Physical Education of Gafsa, University of Gafsa, Gafsa, Tunisia; ^4^ Department of Biostatistics and Medical Informatics, Inonu University Faculty of Medicine, Malatya, Türkiye; ^5^ Higher Institute of Sport and Physical Education of Ksar-Said, University of Manouba, Manouba, Tunisia; ^6^ Tunisian Research Laboratory “Sports Performance Optimization”, National Center of Medicine and Science in Sports (CNMSS) LR09SEP01, Tunis, Tunisia; ^7^ Department of Teacher Education, NLA University College, Oslo, Norway

**Keywords:** molecular hydrogen, pre-exercise ingestion, maximal aerobic speed, time to exhaustion, jumping tests

## Abstract

Molecular hydrogen (H2 gas) dissolved in water to produce Hydrogen-Rich Water. Hydrogen-Rich Water (HRW) is considered as ergogenic aid in different exercise modes. However, acute pre-exercise HRW ingestion effect is unclear regarding athlete performance. This study aimed at investigating acute effect of HRW ingestion on aerobic and anaerobic exercise performance. Twenty-two male amateur middle-distance runners volunteered to participate in this study. In a randomized, double-blind study design, all players ingested 500 mL of HRW or placebo (PLA) supplement 30 min before the start of the tests. Over 4 days, maximal aerobic speed of Vameval test (MAS), time to exhaustion at MAS (Tlim), squat jump (SJ), counter-movement jump (CMJ) and five jump test (5JT) were evaluated. Also, rate of perceived exertion (RPE) and peak heart rate (HRpeak) were measured during the aerobic tests. For Vameval test, HRW ingestion improved MAS, HRpeak and RPE compared with the placebo condition. For Tlim test, HRW ingestion demonstrated improvements in time to exhaustion, RPE and HRpeak. However, no significant change was observed between HW and placebo conditions in SJ, CMJ, 5JT. 500 mL of HRW can significantly improve HRpeak, time to exhaustion, RPE, with no significant effect on MAS, jumping performance in amateur endurance athletes.

## 1 Introduction

Hydrogen (H) atoms consist of one proton and one electron, which forming two hydrogen atoms produce molecular hydrogen (H_2_). Drinking hydrogen-rich water (HRW), inhaling H_2_ gas, or administering H_2_ through baths are typical strategies to administer H_2_ (Fukai, 2020). Hydrogen-rich water (HRW) remains the most convenient method of ingestion (Ohta, 2011). In fact, HRW as hydrogen-rich water electrolysis is a kind of water applied with cathode and anode to decompose molecule of hydrogen (H_2_) and oxygen (O_2_; Ohta, 2011). Previous research popularly recommended HRW doses varying from 0.8 mg to 5.0 mg or 300 mL–500 mL for hydrogen-rich water ingested 5–30 min before exercise or during recovery (Dong et al., 2022).

Regarding oxidative properties, hydrogen is known to scavenge toxic reactive oxygen species (Ohsawa et al., 2007), induce a number of antioxidant proteins (Buchholz et al., 2011; Kawamura et al., 2020), and may reduce muscle fatigue induced by oxidative stress after physical exercise (Aoki et al., 2012).

As an application in sports science, hydrogen-rich water (HRW) has been popularly implicated through different exercise modes such as strength exercise (Javorac et al., 2019; Botek et al., 2021), repeated sprints (Botek et al., 2022), anaerobic activity (Timón et al., 2021) and endurance exercise (Da Ponte et al., 2018; Botek et al., 2019; LeBaron et al., 2019), in trained (Aoki et al., 2012; Da Ponte et al., 2018; Timón et al., 2021) and untrained individuals (Botek et al., 2019; 2021; Timón et al., 2021).

In this context, contradictory results on the effect of HRW on physical performance were documented. In fact, the majority of studies indicate that the ingestion of acute or repeated HRW dose consumed before short-term exercise failed to improve strength (Aoki et al., 2012), repeated sprint times (Brandt-Talbot et al., 2017) or jump performance (Dobashi et al., 2020). However, other studies showed that HRW supplementation enhanced running efficiency at maximal aerobic speed (Aoki et al., 2012; Botek et al., 2019). In addition, studies have shown that acute HRW ingestion before intense physical exercise leads to a reduction in RPE (Botek et al., 2019), lactate concentration (Botek et al., 2019) and stimulated activity of the prefrontal cortex (Hong et al., 2022).

Research also showed that acute ingestion of HRW promoted a post-exercise hypo-lactic effect (Aoki et al., 2012; Ostojic and Stojanovic, 2014) and reduced muscle soreness after strength exercise (Kawamura et al., 2020; Valenta et al., 2022).

The recent review by Zhou et al. (2023) reported several important factors that may contribute to the effects of H2 supplementation on fatigue such as the qualities of the players, the training level, the period of H2 consumption and also the type of exercise.

To date, it is unresolved whether HRW impacts on physical performance and physiological outcomes. Consequently, the main aim of the current study was to examine the acute effect of hydrogen-rich water on physical, perceptual and cardiac responses during aerobic and anaerobic exercises in adult male young men. Based on previous findings (Ostojic and Stojanovic, 2014; Valenta et al., 2022), we hypothesized that 500 mL of HRW ingestion 30-min before exercise fail to improve physical performance or any other physiological variable compared with placebo condition.

## 2 Materials and methods

### 2.1 Participants

**FIGURE 1:**
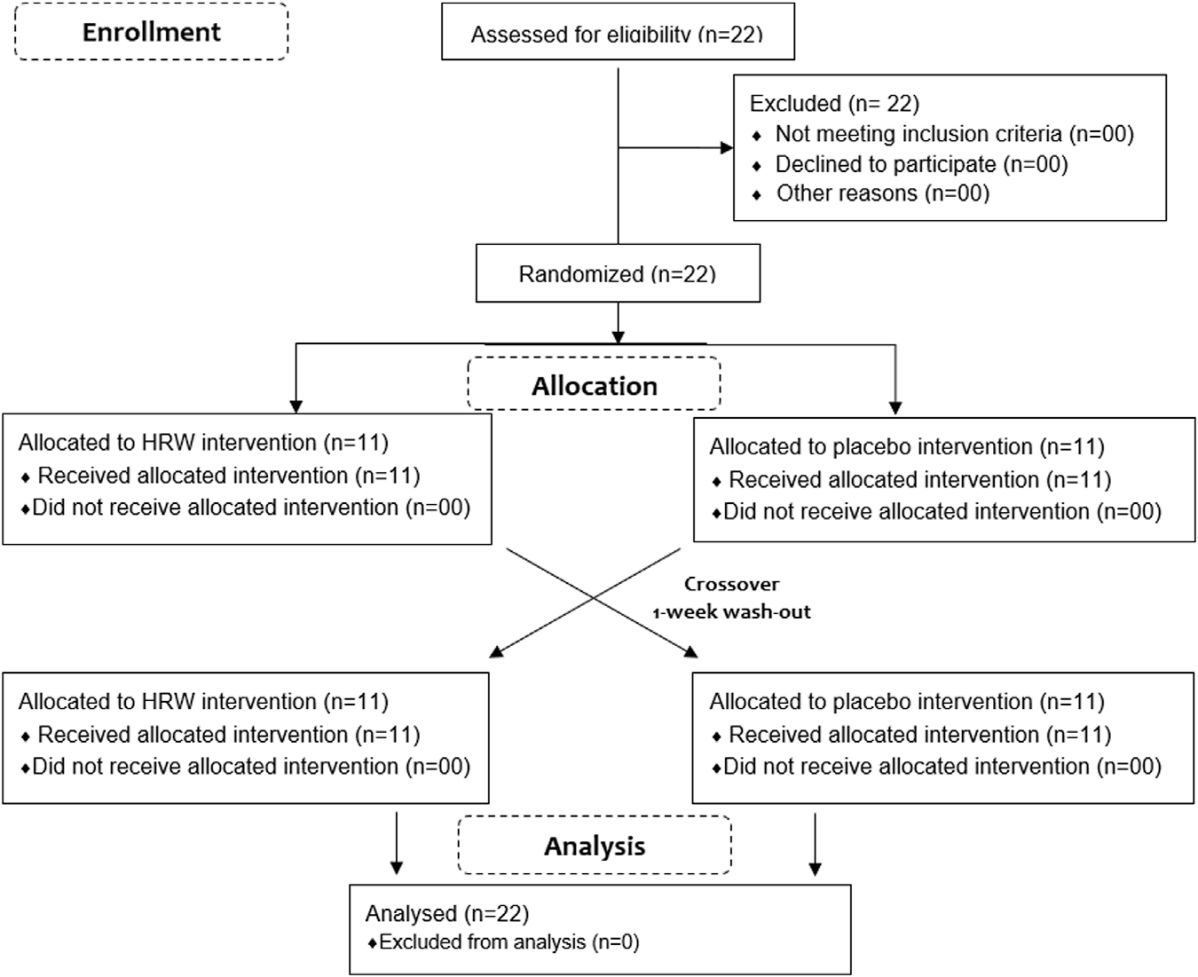
CONSORT flow diagram. Note: HRW: hydrogen-rich water.

G*Power was used to estimate the sample size (Version 3.1.9.2, University of Kiel, Kiel, Germany). The analysis by means of the Student’s t-test of the dependent means revealed that, with this design, 22 players would be required to prove 80% power with a significance level of 0.05 and to detect an effect size d = 0.55. Accordingly, 22 amateur active students (age 21 ± 1 year, body mass 66.85 ± 9.51 kg, body height 1.74 ± 0.10 m and BMI 21.89 ± 1.75 kg m^-1^) volunteered to participate in this study. Players completed a training history questionnaire, which revealed that they had been previously involved in athletics sport, specifically in middle distance running, for 4.18 ± 1.11 years. Prior to participation, the players underwent a medical examination in High Institute of Sport and Physical Education of Kef. Also, none of the players reported having neuromuscular or musculoskeletal disorders affecting the ankle, knee or hip joints before the experimental protocol. All players successfully completed this study (Figure 1). Before providing written and informed consent to participate, players were informed of the potential risks, benefits and following dissemination of the research. High Institute of Sport and Physical Education Ethics Committee approved the study (19 September 2019; UR22JS01/ISSEP-015-19) in accordance with the Helsinki Declaration. All players had to provide written informed consent.

### 2.2 Experimental design

The week preceding the experimental protocol, all test procedures, instruments and equipment were familiarized and put into practice. After the anthropometric measures and familiarization procedures, players were required to complete tests in a randomized, placebo-controlled and double-blind design. Players pre-ingested 500 mL bottle of HRW or placebo during 2 days, with at least 48-h between testing days. During the first day, squat jump test, countermovement jump test and Vameval test were performed. During the second day time to exhaustion and jump tests were performed. After a 1-week washout period, each player was transferred to the other supplementation group for a second investigation period.

Before each testing day, players completed a standardized 10-min warm-up, consisting of 4 min of jogging, lateral moves, dynamic stretching, followed by 2 min of passive recovery.

**FIGURE 2:**
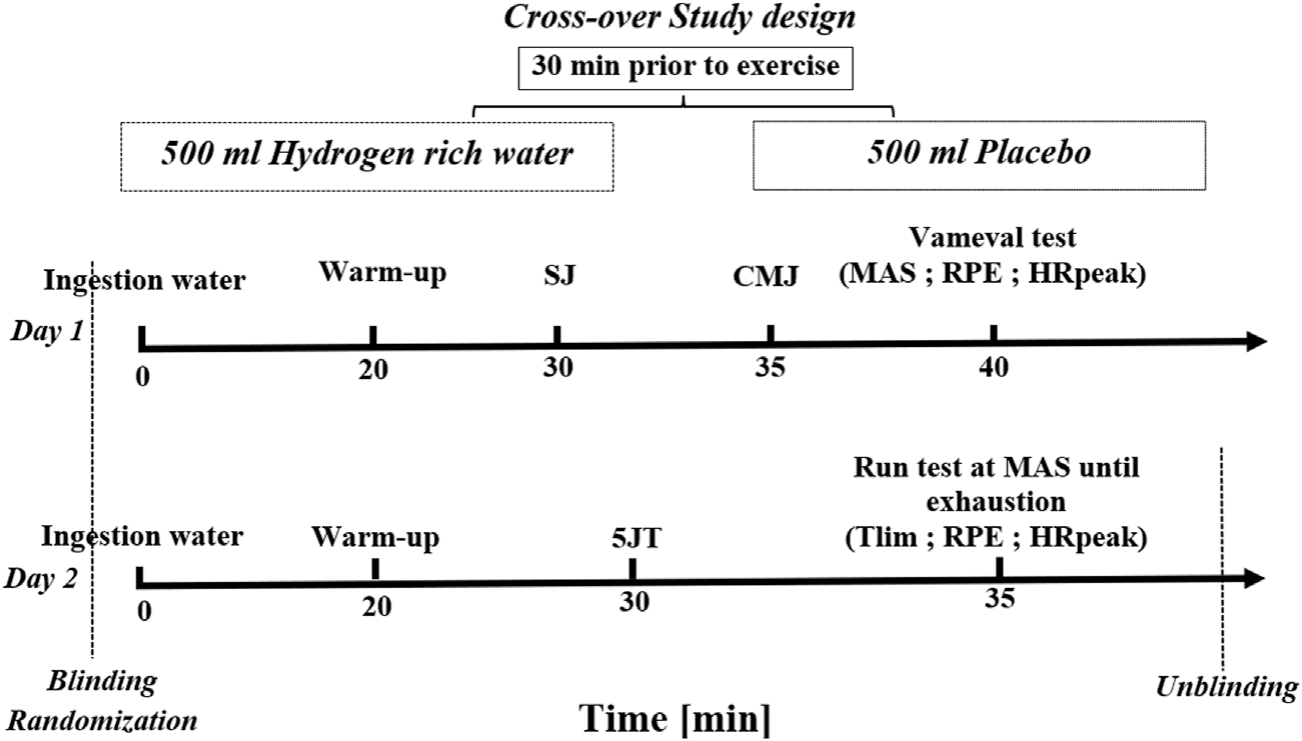
Study design. Note: SJ: squat jump; CMJ: countermovement jump; 5JT: five jump test; MAS: maximal aerobic speed; Tlim: time to exhaution test; RPE: Rate of Perceived Exertion; HRpeak: peak heart rate.

All tests were performed at the same time of day (14 ± 1 h) for each player to avoid any influence by circadian variation. Players followed the same food diaries and water volume ingestion protocol 24 h before each experimental day. All tests were carried outdoors under standardized conditions. The temperature was evaluated between 24°C and 26°C, with relative air humidity above 50% and wind speed <2 m/s. Players were instructed to abstain from using any nutritional supplements or performance aids that might affect their performance, alcohol, and strenuous physical exercise 48 h prior to each testing day. Figure 2 provides an overview of the timing of randomization, the treatment plan and the data collection.

### 2.3 Procedures

In a double-blind manner, players ingested randomly 500 mL of either hydrogen-rich water (HRW) or placebo water (PLA) using the same mineral water (pH: 7.4) and same water temperature (12°C). Players consumed water 30 min before each testing. There was no noticeable difference in taste between HRW and PLW among the players.

The study was randomized and blinded by an independent researcher who was not involved in data collection to ensure the double-blind study design. Using a Latin Square model (Mason et al., 2003) and Research Randomizer, players were assigned to each experimental condition at random (www.randomizer.org). The same investigators carried out all the measurements during the experimental protocol. Additionally, the investigators were also blinded to the water substance (HRW or PLA).

### 2.4 Administration of hydrogen-rich water

HRW was produced by y portable bottle of 550 mL capacity (HEADROGEN HEALTH, Australia). Using this device, water is divided into hydrogen and oxygen gases during an electrolysis process in a time period of 3 min. This hydrogen gas infuses into the water, creating high-concentration hydrogen while removing other gases, particularly chlorine.

Hydrogen concentrations in water were also detected using a hydrogen needle sensor (DHS-001, ABLE, Tokyo, Japan) to verify the operation of the “HYDROGEN HEALTH” device. During drinking, the hydrogen concentration was maintained between 0.55 mmol and 0.65 mmol.

HRW is the high safety profile for the human body and has no side effect (Saitoh et al., 2010) and this device is certified and in compliance with the European Union commercial market (Europe) and FCC (North America) regulations.

### 2.5 Vameval test and time to exhaustion

A 400-m outdoor running track was used for the Vameval test. As a mark, twenty cones were placed every 20 m on the track. The test began at 8.5 km/h^-1^ and increased by 0.5 km/h^-1^ every minute until exhaustion (Cazorla, 2003). At 20-m intervals, players adjusted their running speed to the audio beep. The test ended when the player was unable to maintain the required running speed dictated by the audio beep for two consecutive times. MAS denoted the final completed level and was used for further analysis. The reproducibility of tests was determined using pilot data from 22 players collected during familiarization trials (Vameval test: ICC = 0.994, 95% CI: 0.985-0.998; time to exhaustion at VO2max: ICC = 0.983, 95% CI: 0.959-0.993).

After 48h, a second test of Vameval was adjusted to an intensity corresponding to 100% of the MAS, initiating the race with MAS until voluntary exhaustion (Tlim). Tlim at VO2max has been reported to be variable between subjects (3-8 min; Billat et al., 1994). During tests, the peak heart rate (HRpeak) values was evaluated during both tests using a heart rate monitor (Polar team 2, Polar Electro Oy, Finland). Just after tests, rate of perceived exertion (RPE) was determined using Borg’s RPE (6–20) Scale (Borg, 1982). The 20-point of the RPE scale, ranged from 6 (“very, very light”) to a 20 (“very, very hard”), was used to verbally rate perceived exertion after the tests. Each player responded to the question: “How did you perceive your exertion in your full-body during the test?”. Previous studies have recommended this scale to measure RPE after exercise (Jebabli et al., 2022; Jebabli et al., 2023).

### 2.6 Squat jump and countermovement jump tests

For the squat jump (SJ), players performed a vertical jump from a “squat” position (90° knee flexion angle). Throughout the test, they were instructed to jump as high as they could with both hands around their waist while keeping their chest upright. Players were also told to keep their knees straight during the landing phase.

Players performed a vertical jump from a standing position with an eccentric downward and concentric action for the counter-movement jump test (CMJ). Throughout the test, they were instructed to jump as high as they could with both hands around their waist while keeping their chest upright (Markovic et al., 2004).

The angle of the leg flexion was freely chosen by the players. Additionally, players were instructed to keep their knees straight during the landing phase.

Jumping performance was evaluated with an infrared jumping system (Optojump Next instrument, Version 1.3.20.0, Microgate, Bolzano, Italy) interfaced with a microcomputer. Jump height was calculated for both tests. Players were free to choose the angle of leg flexion. Players were also told to keep their knees straight during the landing phase. No verbal encouragement was provided during the two tests.

The reliability of tests was determined during familiarization trials using pilot data from 22 players collected on two different days (SJ: ICC = 0.958, 95% CI: 0.899-0.983; CMJ: ICC = 0.937, 95% CI: 0.853-0.974).

### 2.7 Five-jump test

The five-jump test (5JT) consists of 5 consecutive strides with feet together at the start and end of the jumps (Chamari et al., 2008). From the starting position feet together, the player jumps directly forward with one leg of his choice, alternating the left and right feet 2 times each, and completes the fifth stride with feet together.

The front edge of each player’s feet in the starting position and the back edge of their feet in the end position were measured with a tape measure to determine their 5JT performance. No verbal encouragement was provided during the test.

The reliability of tests was determined during familiarization trials using pilot data from 22 players collected on two different days (5JT: ICC = 0.958, 95% CI: 0.899-0.983).

### 2.8 Statistical analyses

Normal data distribution was tested and confirmed using the Shapiro Wilk test. Accordingly, data were presented as means and standard deviations (SDs). Test re-test reliability of the variables was assessed using Cronbach’s model of the intraclass correlation coefficient (ICC) and 95% confidence intervals (95%CI).

Appaired T-Student was performed for MAS, Tlim, HRpeak, RPE, SJ, CMJ and 5JT. As effect sizes, Cohen’s d was calculated to quantify meaningful differences in the data with demarcations of trivial (<0.2), small (0.2–0.59), medium (0.60–1.19), large (1.2–1.99) and very large (≥2.0; Chow, 1988). In addition, we plotted percentage changes between conditions in each parameter using the following formula: Δ (%) = ([T2mean/T1 mean]-1) * 100. The ICC between the familiarization trials was interpreted having as poor (<0.5), moderate (0.5–0.75), good (0.75–0.9), and excellent (>0.9) reliability based on the lower bound of the 95% confidence interval (CI) (Koo and Li, 2016).

Statistical significance was accepted, *a priori*, at *p* < 0.05. Data were analyzed using the SPSS 22 package (SPSS Inc., Chicago, USA).

## 3 Results

### 3.1 Vameval test and time to exhaustion

**FIGURE 3:**
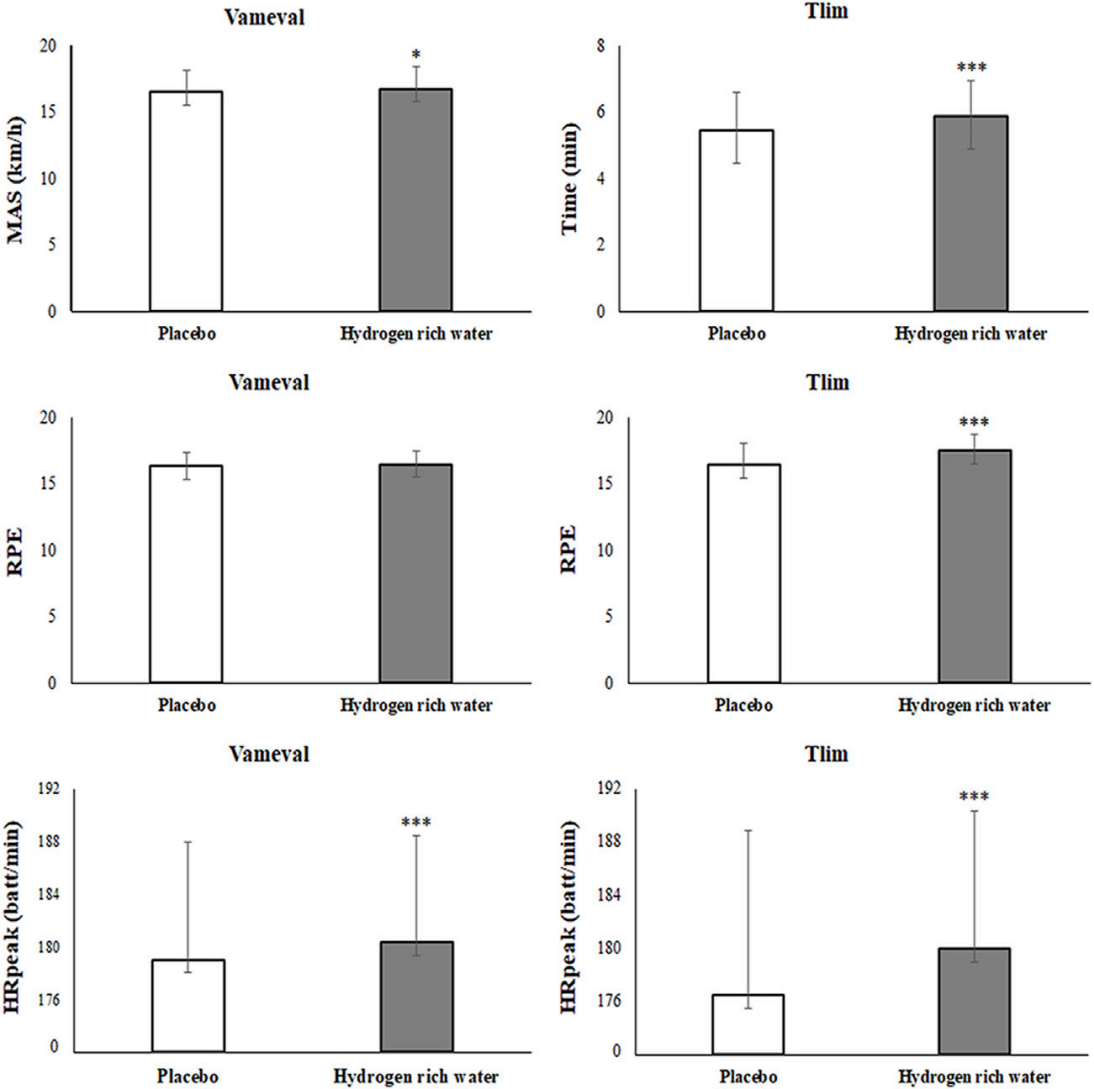
Effect of hydrogen rich water compared to placebo on aerobic performances. Note: Mean ± SD; MAS: maximal aerobic speed; Tlim: time to exhaustion at MAS; RPE: rate of perceived exertion; HRpeak: peak heart rate.

For Vameval test, HRW ingestion demonstrated a significant improvement of MAS (*p* = 0.04; ∆ = 0.55%; d = 0.06), HRpeak (*p* < 0.001; ∆ = 1.01%; d = 0.21) and RPE (*p* < 0.001; ∆ = 0.83%; d = 0.14) compared to the placebo condition (Figure 3).

For Tlim test, HRW ingestion demonstrated significant improvements of time to exhaustion (*p* < 0.001; ∆ = 7.71%; d = 0.39), RPE (*p* < 0.001; ∆ = 6.65%; d = 0.77) and HRpeak (*p* < 0.001; ∆ = 1.98%; d = 0.31) compared to the placebo condition Figure 3).

### 3.2 Jump tests

No differences were reported between HW and placebo conditions in SJ (*p* = 0.120; ∆ = 2.26%; d = 0.10), CMJ (*p* = 0.382; ∆ = 1.62%; d = 0.07) and 5JT (*p* = 0.267; ∆ = 0.57%; d = 0.04; Table 1).

**TABLE 1:** Effect of hydrogen rich water compared to placebo on jumping performances.

	PLA	HRW	∆(%)	*p*-value	d
**SJ (cm)**	27.35 ± 6.09	27.96 ± 6.45	2.26	0.120	0.10
**CMJ (cm)**	28.90 ± 6.70	29.36 ± 7.01	1.62	0.382	0.07
**5JT (m)**	10.54 ± 1.50	10.48 ± 1.53	0.57	0.267	0.04

Note: *. Significance of simple effect between PLA, and HRW. PLA: placebo water; HRW: Hydrogen-rich water; ∆ (%): mean difference; d: effect size; SJ: squat jump; CMJ: countermovement jump; 5JT: five jumps test; HR: heart rate; RPE: rated perceived exertion.

## 4 Discussion

The primary aim of this study was to assess the physical, physiological and perceptual responses after consumption of 500 mL of HRW. Based on previous studies that demonstrated an anti-fatigue effect of HRW consumption before exercise without having an ergogenic effect on physical performance (Ostojic and Stojanovic, 2014; Valenta et al., 2022), we have hypothesized that HRW consumption before exercise would have no ergogenic effects on physical performance. Contrary to our hypothesis, we observed a significantly beneficial effect of HRW on MAS and time to exhaustion during maximal aerobic speed exercise. However, no significant effect of HRW on jumping performance was observed.

The current findings suggest that the pre-exercise administration of 500 mL HRW elicited a significant enhancement in time to exhaustion and MAS. In agreement with the present study, Mikami et al. (2019) reported an improvement in endurance capacity, as judged by estimated VO2max following 500 mL of H_2_-water ingestion. However, studies have demonstrated that HRW had no significant effects on maximal (Valenta et a., 2022) and submaximal (Ito et al., 2020; Ooi et al., 2020; Alharbi et al., 2021) exercises performance using different HRW characteristics. As example, previous research concluded that acute doses of HRW (290 mL or 2.0 mL⋅kg-1) 10 min before running or cycling to exhaustion did not improve performance trained athletes (Ito et al., 2020; Ooi et al., 2020).

Therefore, it is necessary to carefully study the variation of different characteristics of HRW such as the dose number, volume, concentration and moment of ingestion. In fact, a single dose of H2 immediately before exercise, for players who had not previously consumed H2, promotes a greater reduction in fatigue in healthy adults, compared to the longer period of implementation of the H2, or multiple H2 inputs (Zhou et al., 2023). One possible explanation is that H2 appears to be a neuroprotective agent that facilitates the restoration of neuronal oxidative damage (Zhou et al., 2023). Also, Mikami et al. (2019) reported that more than 59% of H2 can be exhaled within the first hour of taking H2.

As part of the present study, these factors were controlled. Furthermore, our results are contained by a sufficient sample size and high measurement reliability compared to previous studies (Aoki et al., 2012; Botek et al., 2019) having small sample sizes (8–12 participants) and who did not find an ergogenic effect of HRW consumption.

According to previously published studies, the increase in aerobic capacity during the consumption of HRW is difficult to explain physiologically with other studies because it is difficult to compare our results to other studies that have not carried out the same methodology and the same doses or concentration of HRW. However, one possible mechanistic explanation for the benefit of HRW pre-ingestion on aerobic performance is that H2 appears to be a neuroprotective agent that facilitates the restoration of neuronal oxidative damage (Zhou et al., 2023). Also, hydrogen-water has been shown as natural antioxidant to reduce and remove hydroxyl radical reaction with the decrease in blood lactate and muscle pH during in strenuous physical exercise (Aoki et al., 2012; Nogueira et al., 2021) which improve muscle function, and alleviated muscle pain perception (Botek et al., 2021).

The considerable effect of H_2_ on metabolism might also explain partly these results, since H_2_ maintains in the lipid and glycogen phases for a longer period (Alharbi et al., 2021; Nogueira et al., 2021). The H2 antifatigue effects, mediated via enhanced metabolic coordination and immune redox balance, specifically through increased liver glycogen storage, lactate dehydrogenase and glutathione peroxidase activity; reduction of interleukin-6, inter-leukin-17 and tumor necrosis factor-α might also explain the performance enhancement (Iuchi et al., 2016).

Although we did not assess oxidative stress markers, we must mention that these hypotheses are highly speculative. Future research is therefore needed to examine the effect of acute HRW on oxidative stress markers before and after exercise.

Regarding heart rate variation, ingestion of HRW significantly improve HRpeak during both Vameval and Tlim tests compared to the PLA condition. These data are in discrepancy with the previous studies of acute hydrogen water (LeBaron et al., 2019; Ooi et al., 2020; Hong et al., 2022). In fact, LeBaron et al. (2019) showed no significant change of HRpeak after the ingestion of 5 mg HRW during a graded treadmill exercise test to exhaustion. In addition, Ooi et al. (2020) studied the impact of ingestion of HRW on maximal treadmill running in endurance trained athletes. They showed that 290 mL HRW showed no significant difference for HRpeak. At the same, no significant effect of pre-exercise administration of 1,260 mL HRW on HRpeak in runners (Hong et al., 2022). The discrepancy in HRpeak values during maximal exercises between our study and the other studies may be explained by the difference in participant type (amateur active student; endurance-trained runners), exercise type (Vameval test; incremental treadmill running; cycling exercise), the dose of H2-water (1 dose of 500-mL; 2 dose of 290-mL; 20-min H2 gaz respiration). In this context, it is unclear whether HRW increase HRpeak during maximal exercise. However, ingestion of HRW, in some cases, may increase circulating epinephrine levels, which could increase maximum heart rate (Adzavon et al., 2022).

HRW heart rate lowering observed in our study might be explained by the anti-inflammatory and anti-apoptotic effects of hydrogen in myocardial cells (Kamimura et al., 2011). In fact, hydrogen exerts myocardial protection from heart diseases via inhibiting oxidative injury, apoptosis and cytokines release (Ara et al., 2018).

Our results showed that HRW causes a significant improvement of the RPE value during Tlim test compared to the PLA condition. This finding is logical in nature as the participant’s perception of effort would be maximal if the cardiovascular system was working harder (Cazorla, 2003). Additionally, this result could be explained by the fact that the participants are active and have not previously performed tests at maximum intensity until exhaustion.

These findings are in disagreement with previous findings by Botek et al. (2019), which reported a lower RPE during a steplike incremental exercise after ingesting 600 mL of HRW.

Taken together, future studies considering the relationship between RPE and physiological variables (heart rate, lactate, *etc.*), after HRW ingestion, are required.

Regarding to anaerobic exercises, our results showed no significant difference between condition HRW and condition PLA in SJ, CMJ and 5JT. To our knowledge, only one study has examined the effect of hydrogen-rich water ingestion on physical performance in vertical jumps. Indeed, Dobashi et al. (2020) observed that the ingestion of 500 mL HRW during 3 days just before the exercise showed no significant difference in the physical performance of the jumps which is in agreement with our results. However, Dong et al. (2022), reported that acute ingestion of HRW (1.6 ppm), during 7 days, is an appropriate means of hydration to effectively improve lower power performance during dragon boat exercise.

Moreover, the study of Timón et al. (2021) showed that chronic HRW intake for 1 week had a positive effect on the performance in the anaerobic test with an increase in peak power and mean power and a decrease in the fatigue index the performance of trained cyclists during the anaerobic test. Therefore, a potential reason for the lack of an apparent effect of the lower explosive power may be the HRW ingestion characteristic (dose number, concentration, volume, timing).

This research has several limitations. The present study involved only the acute effect on HRW before tests. So, the results cannot be generalized with the results of chronic ingestion of HRW. Added to this, oxidative stress markers were not evaluated, in this research, which may be helpful for a deeper understanding how HRW improve performance responses. Also, Although the students were well familiar with the RPE scale (6-20), we observed that the RPE values after maximal exercise did not reflect work at maximum intensity (RPE<19; HRpeak close to theoretical maximal HR) raising the question of the reasons for this low value in active participants. Therefore, future studies will require continuous assessment of RPE during exercise in relationship with heart rate variation followed by an assessment of blood lactate concentration post exercise.

## 5 Conclusion

This study found that a dose of 500 mL HRW intake 30-min before exercise seems to be an effective hydration strategy, although a greater MAS, time to exhaustion, peak heart rate and RPE without any change in jumping performances.

## Data Availability

The original contributions presented in the study are included in the article/Supplementary Material, further inquiries can be directed to the corresponding authors.
